# Omics era in forensic medicine: towards a new age

**DOI:** 10.3906/sag-1912-197

**Published:** 2020-08-26

**Authors:** Ramazan AKÇAN, Burak TAŞTEKİN, Mahmut Şerif YILDIRIM, Halit Canberk AYDOGAN, Necdet SAĞLAM

**Affiliations:** 1 Department of Forensic Medicine, Faculty of Medicine, Hacettepe University, Ankara Turkey; 2 Department of Forensic Medicine, Faculty of Medicine, Afyonkarahisar Health Sciences University, Afyonkarahisar Turkey; 3 Department of Nanotechnology and Nanomedicine, Graduate School of Science and Engineering, Hacettepe University, Ankara Turkey

**Keywords:** Forensic medicine/science, omics, post-mortem interval, drugs of abuse

## Abstract

**Background/aim:**

Forensic medicine and sciences is a multidisciplinary branch of science, which frequently benefit from novel technologies. State of the art omics technologies have begun to be performed in forensic medicine and sciences, particularly in postmortem interval, intoxication, drugs of abuse, diagnosis of diseases and cause of death. This review aims to discuss the role and use of great omics (metabolomics, proteomics, genomics and transcriptomics) in forensic sciences, in detail.

**Materials and methods:**

A detailed review of related literature was performed, and studies were subdivided as per the type of omics.

**Results and conclusion:**

Omics seems as a revolutionary step in forensic science and sure carries it towards a new age. The number of forensic studies utilizing omics steadily increases in last years. Omics strategies should be used together in order to gather more accurate and certain data. Additional studies need to be performed to incorporate omics into routine forensic methodology.

## 1. Introduction

The topic “forensic medicine and sciences” is a subdivision of science which is described as the practice of medical/paramedical scientific knowledge to establish facts in civil and criminal law [1]. Forensic medicine has a multidisciplinary structure in collaboration with other areas such as forensic chemistry, toxicology, biology, psychiatry, and genetics, etc. Since forensic sciences share a common scientific methodology with other science disciplines it is open to collaborate or benefit from novel technologies appeared in any scientific discipline. Therefore, omics emerges as a revolutionary step in forensic sciences and sure carries it towards a new age. The publications regarding omics in various disciplines have become a rising scientific trend worldwide, during the last decade. Furthermore, the number of forensic studies utilizing the methodology of omics has enormously increased in last years. In order to draw attention of forensic and medical professionals, and encourage new studies; the roles and benefits of omics in forensic medicine and sciences are discussed in this review.

## 2. Omics

Omics is a novel multidisciplinary field of various scientific methodologies. Omics defines the collective technologies that measure some characteristics (roles, relationships, and actions) of certain molecules like metabolites, proteins and genes, ending in -omics, as in metabolomics, proteomics, genomics, etc. In past years, omics technologies were applied to diverse fields, because of the ability to assess little changes in a large-scale data with exhaustive metabolite evaluation [2]. With the development of technology, omics-based strategies become cheaper, faster, and very informative, and offer potent alternatives to conventional technics. Furthermore, sample selection has a key role to success in target study. Various human samples can be used for analysis such as blood [3,4], urine [5], hair [6,7], nail [8–10], faeces[11,12], aqueous humour [13] etc. The interactions of omics with each other have been shown in Figure.

**Figure:**
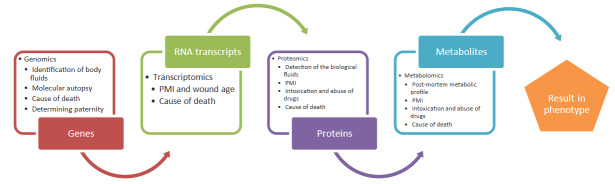
General scheme of relationship between omics and phenotype.

### 2.1. Metabolomics

#### 2.1.1. Definition of metabolomics

Metabolomics comprises the studies that are focused on assessment of metabolites and related markers of an organism. Metabolomics is described as omics of the minor molecules, called metabolome, due to the low molecular weight of metabolites (<~1000 Da) such as amino acids, etc [14]. The word “metabolomics” can be attributed to Fiehn, who described the field in 2002 as a comprehensive and detailed analysis all of the metabolites in an organism.

The metabolomic study procedure consists of processes such as gathering sample, preparation of the samples, analysis, obtaining and interpreting data, respectively and also supplies specific opportunities to understand the status of organisms related to the metabolome [15]. Different metabolomics-based strategies are used for sample analysis methods like gas chromatography/mass spectrometry (GC/MS), liquid chromatography/mass spectrometry (LC/MS), Fourier transform infrared spectroscopy (FTIR), Raman spectroscopy (RS), direct injection mass spectrometry (DIMS), nuclear magnetic resonance (NMR), etc. Recently, metabolomics has an increasing popularity in forensic medicine due to comprise faster and reproducible methods and picturing biological status of the organism [16].

#### 2.1.2. Role of metabolomics in forensic medicine

##### 2.1.2.1. Postmortem interval (PMI)

Joshua Lederberg was first who describe the term “microbiome” as the “ecological community of microorganisms in human body” [17]. Most of the endogenous mammalian microorganisms live in the gastro-intestinal tract. After death, the microbiome causes bloating and, eventually, rupture of corpse [18]. After the death of host organisms, bacterial community rapidly turn into complex communities [19]. Furthermore, these communities begin to change the biochemical and metabolic profiles of body specimens. Metabolomic methods based on such profiles can supply important data about the postmortem interval and cause of death, if convenient markers are detected after death [20].

The use of metabolomics in PMI is not limited to the microbiome. In 2009, Hirakawa et al. [21] investigated metabolic alterations after death in rat muscles by proton (1H) NMR spectra. The results of the study suggested that the metabolic profiles of the tissues varied in accordance with the manner of death, and correlated with PMI, as well. Consequently, the authors reported that NMR-based metabolic profiling could supply beneficial data about the estimation of PMI and cause of death.

Not only NMR was useful for achieving postmortem metabolic profile, MS methods are also highly convenient in this area [14, 22–27]. In 2005, Sato et al. sacrificed 36 rats by suffocation, collected their blood at certain times of postmortem period and detected 70 metabolites using GC-MS/MS [26]. The authors identified diverse endogenous metabolites altered with time since death. Thus, plasma metabolic profiling succeeded to predict PMI in the presence of some conditions. Researchers from Seoul combined ultra performance liquid chromatography/quadrupole time-of-flight mass spectrometry (UPLC/Q-TOF MS) based metabolomics in order to analyze metabolite changes in rat livers related to the PMI, in 2012 [27]. After acquiring the data, statistical methods were performed to detect the significantly increasing and decreasing metabolites which allowed to classify samples for PMI prediction.

##### 2.1.2.2. Intoxication and abuse of drugs

Intoxication and abuse of drugs have become an international public health problem, particularly with increasing numbers of new substances entering the drug market. Holmes et al. [28] introduces a new trend “xenometabolome” and describe it as “the multivariate description of the xenobiotic metabolite profile of an individual or sample from an individual that has been exposed to drugs through any route, environmental pollutants or dietary components that cannot be completely catabolized by endogenous metabolic enzyme systems”, in 2007. They had used routinely detected drugs in urine and utilized statistical methods to determine relations of metabolites. Their method achieved quick identification of drug metabolites. Thus, their study paved the way in forensic medicine by considering all the possible pharmacokinetic of a drug, which might be used to find out the time of intake of drug and PMI. Shima et al. [29] used MS-based metabolomics to determine the relationship of fundamental intoxication mechanism and metabolic networks on urine and plasma in 2011. The authors conducted this study by sacrificing 24 methamphetamine-intoxicated rats. The results of the study provided an explanation for main mechanism of various effects of intoxication. Additionally, intoxication process was objectively demonstrated by using biological fluid-based diagnostic and forensic methods. In 2013, Tsai et al. used UPLC/Q-TOF MS based metabolomics for screening and confirmation of a total of sixty-two drugs of abuse and their metabolites in urine [5]. They achieved much better accuracy by using UPLC/Q-TOF MS than conventional immunoassay methods.

Besides, urine and blood, hair and nails can also be used in the metabolomics experiments in order to detect metabolites abused drugs or exposed toxic agents. The detectability time of metabolites in hair and nails is relatively long, typically months in hair and months/years in nails. When the drug and its metabolites have been removed from commonly analyzed body fluids such as urine or blood, long detection time makes metabolomics the most convenient alternative for interpretation of forensic cases. A study by Kim et al. [7] aimed to develop a new strategy for hair analysis in drug-facilitated crime cases by UHPLC-MS/MS, in 2016. They injected a mixture of zolazepam and tiletamine, and shaved 10 rats. Five weeks after the injection, they collected the hair of rats. Results of their study showed that even after a single exposure detection of the drugs is feasible in study objects, which can consequently be performed in crime cases. Krumbiegel et al. [30] collected nails from 70 postmortem cases and analyzed samples by LC/Q-TOF MS, in which eighty-nine different analytes have been detected in specimens. In conclusion, they suggested that when the enough amount of hair is not available, using nail samples provides comparable results in point of determining the long-term exposure.

An up-to-date review dealing with various implementations of metabolomics methods for determination of abuse of drugs based on biomarker research was published in 2019. It is also a summary of present information about metabolomic methods used for possible biomarkers displaying abuse [31].

##### 2.1.2.3. Diagnosis of disease and cause of death

The metabolomics have ability to reveal a clear picture of metabolic profile and status of organisms and also can be utilized for diagnosis of a disease or cause of death. Metabolic profiles have been established for certain diseases such as Alzheimer’s disease [32], Parkinson’s disease [33], preeclampsia [34], intrauterine growth restriction (IUGR) [35], anaphylaxis [36, 37], and etc.

Ghauri et al. obtained cerebrospinal fluid from 12 autopsy cases of neuropathologically confirmed Alzheimer’s disease and detected reduction in citrate levels in comparison of control samples by 1H NMR spectra [38]. 

The metabolomics has facilitated monitoring of metabolic changes in anaphylaxis and understand its pathophysiological processes in 2012 [37]. Hu et al. aimed to obtain metabolic profile of anaphylaxis in animal models and search for possible markers via GC-MS. The authors achieved to determine major metabolic alterations and detected the metabolites associated with “energy metabolism and signal transduction in anaphylaxis”.

Based on abovementioned scientific suggestions and facts metabolomics can be an alternative and a new method for diagnostics and establishing the cause of death.

### 2.2. Proteomics

#### 2.2.1. Definition of proteomics

Proteome contains totally more than 100.000 expressed protein in a cell of organism, which can be affected by environmental conditions and gene activity. Proteomic approach studies on the proteome. The word “proteomics” was first described by Marc Wilkins in 1995 as an entire organism’s protein complement. His idea was screening and determining all the proteins produced by the DNA of an organism [17]. Proteomic markers are related to the phenotype, so it takes an advantage compared to genomics and transcriptomics markers. Proteomics compasses the determination of amino acid sequence, modifications, structure and possible pathways in the cell. Various proteomics-based strategies such as enzyme-linked immunosorbent assay (ELISA), mass spectrometric immunoassay (MSIA), matrix-assisted laser desorption/ionization (MALDI), surface-enhanced laser desorption/ionization (SELDI), LC-MS/MS, and etc. can be used [39–42]. Proteomics-based methods allow many applications in forensic medicine, such as the determination origin of biological samples without any damaging effect to DNA [43]. 

#### 2.2.2. Role of proteomics in forensic medicine

##### 2.2.2.1. Detection of the biological fluids

The proteomics-based strategies have been developed to detect and identify the proteins of biological fluids such as blood, saliva, lacrimal fluid, seminal fluid, vaginal fluid, sweat, and urineon a surface during scene investigation or on a body part. These strategies are applicable without any destruction of the DNA and it is the most important advantage of this method. In a crime scene, firstly presumptive tests can be used, such as luminol for blood, specific light sources for saliva and semen. In the next step, these traces need to be verified via immunochemical methods or ELISA. On the other hand, MS-based proteomic approaches introduced to detect the origin of specimens and identification of body samples. A review was published about analysis of body fluids by Virkler and Lednev in 2009 and summarized all current and older tests to detect human body fluids as a list [43].

##### 2.2.2.2. Postmortem interval

Pittner at al. [44] tried to detect PMI with a novel approach, postmortem degradation of skeletal muscle proteins in 2015. For this purpose, they studied in porcine muscles using sodium dodecyl sulfate-polyacrylamide gel electrophoresis (SDS-PAGE), western blot and casein zymography. Results of study showed that some of the muscle proteins (desmin, titin, nebulin, cardiac troponin-T, and SERCA1) decreased regularly and predictably. In a following study, in 2016, Pittner et al. [45] analyzed human postmortem skeletal muscle samples, which revealed similar results to previously conducted porcine study, and showed a predictable protein degradation processes in human muscle. Recently a study by Choi et al. [46] investigated the ability of usage unbiased protein analysis to find out the postmortem protein changes and detect new possible markers for PMI estimation. They used proteomic profiling of rat and mouse skeletal muscle samples using MS-based proteomic method. Thus, the authors totally analyzed postmortem changes, and consequently they focused on two proteins (eEF1A2 and GAPDH) because of consistently degradation postmortem in both rats and mice. Their results showed that eEF1A2 and GAPDH proteins appeared to be useful indicators for PMI delimitation.

Handke et al. [47] aimed to use bacterial identification as PMI indicators by proteomics-based approach in 2017. The authors placed pieces of pork outside and took surface swabs at six different time points up to 60 days. Proteomic analyses were performed with MALDI-TOF and LC-MS/MS, and they partially achieved the detection of bacteria that colonized the body after various PMI. Shotgun metaproteomics, which brings a new perspective in microbiome research, enables the characterization of both microbial and host proteins simultaneously in terms of microbiome [48].

In a study, Procopio et al. [49] investigated the usability of bone proteomics to estimate PMI in 2018 for the first time. They found several new potential markers, such as biglycan which plays a role in the bone growth and mineralization. In 2019, Prieto-Bonete et al. [50] used 40 femur bones of cadavers and found 48 proteins by proteomic-based approach using LC-MS/MS method to estimate PMI. According to their proteomic profile results, these can only supply a relative estimate of PMI.

##### 2.2.2.3. Intoxication and abuse of drugs

In a study regarding experimental chronic Methylmercury (MeHg) intoxication by intragastric gavage, Bittencourt et al. [51], assessed proteomic profile and analyzed mercury levels in rat salivary glands. Their results showed that the exposure to MeHg has strong relationship with alterations in proteomic profile of salivary glands.

In 2018, a review regarding human hair proteomics in terms of its diagnostic ability and therapeutic potentials was published and critically discussed about identification of possible biomarkers of diseases utilizing hair as a substrate [52]. They suggested that further investigations were needed to establish “a disease-specific human hair proteome database”. 

##### 2.2.2.4. Diagnosis of disease and cause of death

Breakthroughs in “-omics” carry a promising potential to assess risk factors and find out pathophysiological mechanisms in sudden unexplained death (SUD), in early period of life. Broadbelt at al. focused on hypothesis suggesting proteomics might unveil unknown abnormal protein concentrations related to tryptophan hydroxylase (TPH2) and serotonin (5-HT) regulation in sudden infant death syndrome (SIDS) cases using an MS-based proteomic strategy [53]. The authors detected a considerable reduction out of the groups of 14-3-3 signal proteins in the gigantocellularis of the medullary 5-HT system among SIDS cases compared to control cases that were also suffered from decrease in TPH2 and 5-HT levels.

Proteomic studies may be supportive in determining the anaphylaxis related cause of death. In a several studies evaluating postmortem blood concentrations of tryptase and ß-tryptase were detected very high in contrast medium anaphylaxis [54], less in food anaphylaxis [55], and intermediate in insect bites-related anaphylaxis [56]. On the other hand, cases as heroin-related deaths [57], or posttraumatic deaths and hearth diseases [54] were not clearly related to ß-tryptase levels. Eosinophilic cationic protein (ECP) is a basic protein located in the eosinophil and ECP is released during degranulation of eosinophils. But the ECP shows lack of specificity in anaphylaxis because of its high concentrations detected even in deaths of asthma patients and in heroin-related deaths [57, 58]. Consequently, if specific IgE, high blood tryptase levels, high ECP concentrations, and degranulation of mast cells in tissues are evaluated together, the cause of anaphylaxis-related deaths may be established with a high precision.

### 2.3. Genomics

#### 2.3.1. Definition of genomics

The human genome comprises over three billion bases and over 30.000 genes that coding proteins. Genomics studies on the genome of an organism include all of the genome-related functions, as well. Dr. Thomas H. Roderick, a geneticist, was the first to use the term “genomics”, in 1986 [59]. Diversity of genetic material between two people is approximately 1%. This variation makes each of human being different. Various genomic-based strategies can be used such as real-time polymerase chain reaction (RT-PCR), MALDI-TOF, microarray and next generating sequencing.

#### 2.3.2. Role of genomics in forensic medicine

##### 2.3.2.1. Detection of the biological specimens

The analysis of DNA has contributed to matching a suspect to a scene of crime through identification of body fluids. Nakanishi et al. investigated Bacteroides uniformis, Bacteroides vulgatus and Bacteroides thetaiotaomicron in order to identify feces of different individuals by using RT-PCR. They detected the gene sequences of these bacteria in different samples, such as feces, blood, and saliva, etc. The authors detected either Bacteriodes uniformis or Bacteriodes vulgatus in all fecal specimens. Thus, the results showed that if a sample contains Bacteriodes uniformis or Bacteriodes vulgatus, the sample contains feces.

The microbial flora is a significant clue for key parameters of forensic investigations. These genomic approaches in microbial flora considered as extremely promising and will potentially play a more valuable role in the near future’s forensic applications.

##### 2.3.2.2. Postmortem interval

Bacterial community becomes a complex community after the host organisms dies. This “epinecrotic” bacterial communities can be used in estimation of PMI. Pechal et al. [19] discussed the necrobiome, and organisms associated with the decomposition of remains. They detected a significant correlation for overall microbiome as decomposition progressed and developed a statistical model. The authors’ new method of using high throughput metagenomic sequencing has significant potential to estimate PMI in forensic medicine.

##### 2.3.2.3. Intoxication and abuse of drugs

Personalized medicine is a novel approach towards the evaluation, diagnosis and treatment of a patient. It has some features such as personalized, predictive, preventive, participative and precision medicine [60]. Beside personalized medicine, Wong [61] introduced a new term for the first time, personalized justice. According to Wong, personalized justice complements a collaboration of translational and personalized medicine. Therefore, pharmacogenomic and toxicogenomic methods may protect someone from being charged. In accordance with this, Sallaee et al. [62] published a report of nine-years old boy that was on medications such as methylphenidate, clonidine, and fluoxetine due to a number of psychiatric conditions. After 10 months, he developed some complications, such as gastrointestinal toxicity, disorientation, incoordination, and seizures. Later patient died of cardiac arrest following a status epilepticus. Postmortem toxicologic analysis of deceased showed that severalfold higher fluoxetine and norfluoxetine concentrations. Therefore, child’s parents investigated because of possible fluoxetine intoxication. However, pharmacogenomic and toxicogenomic studies of deceased revealed that boy had a poor P450 CYP2D metabolizer genotype, resulting in accumulation of fluoxetine. Thus, child’s parents were acquitted of the charges.

In 2007, Ikematsu et al. [63] exposed rats to toluene and used a PCR method to detect the consequently expressed genes in rat brain. They detected 20 DNAs by toluene inhalation. Their results contributed to show the patho-physiological effects of toluene inhalation on rat brain.

Saito et al. [64] investigated agranulocytosis/granulocytopenia (CIAG) secondary to clozapine-related genetic risk by genomics-based approach, by assessing possible single nucleotide polymorphism (SNP) survey in Japanese population, in 2016. They utilized a genome-wide pharmacogenomic and toxicogenomic analysis to evaluate subjects. Their results revealed “HLA-B*59:01” as a risk factor for CIAG.

##### 2.3.2.4. Diagnosis of disease and cause of death

Implementation of genomics is of high importance in order to find out and assess the etiology/cause and mechanisms resulting in death at molecular-level, which is commonly defined as “molecular autopsy”. Genomics approaches may be added in routine investigation in combination with conventional autopsy, which might be useful for detection of cause and mechanism of death [65, 66]. Cardiac molecular autopsy has ability to provide a physio-pathological basis for sudden unexpected death (SUD). In 2004, Tester et al. [67] performed a molecular autopsy of 49 SUD cases. In their study, a pathogenic cardiac channel mutation detected in 35% of SUD cases. RyR2 mutation was present in 1 of every 7 cases of SUD. In 2006, Tester and Ackerman [65] published a review regarding the key role of molecular autopsy inconventional autopsy-negative SUD cases through focusing on studies dealing with unexplained sudden cardiac death cases underwent a molecular autopsy.

Genomics-based methods are also convenient in the diagnosis and estimation of tendency to fatal thromboembolic and hypertensive cases. Venous thromboembolism (VTE) takes place among the most common causes of SUD. The correlation between SNPs and increased risk for VTE has been comprehensively investigated [68]. SNPs in several genes are responsible for the risk up to 10-fold for hypertension and related clinical events, such as acute coronary syndrome, left ventricular hypertrophy and preeclampsia [69–71]. 

In forensic medicine, genomics-based approach aims to detect the genomic risk of the individual, establish the basis, causes and mechanisms of death, thus provide an evidence-based, unbiased approach to death assessment [72,73]. Furthermore, it is of high importance to feedback decedents’ relatives for the similar genomic risk and direct them to a clinician to onset of appropriate prevention strategies.

### 2.4. Transcriptomics

#### 2.4.1. Definition of transcriptomics

Transcriptome is the whole set of RNA transcripts produced by entire genome. Transcriptomics defines novel technologies focusing on “studying the transcriptome of an organism”, which utilize microarray analyses and PCR as common analytical methods. 

#### 2.4.2. Role of transcriptomics in forensic medicine

##### 2.4.2.1. Postmortem interval

The transcriptomic approach carries a potential to explain cell or tissue viability during the process towards local necrosis or death, and analysis of mRNA has ability to provide quantitative evidence [74]. Oehmichen et al. [75] aimed to detect “synthesis rates of RNA and DNA” from a site of injury. The authors made experimental wounds to rats’ both ears and obtained biopsies intravital and postmortem. As a promising finding, they found that DNA synthesis continues after death, which showed that determination the age of a wound after death. Takamiya et al. [76] studied mRNA expression of basic fibroblast growth factor to detect the age of wound in various wound locations of mice. They found that the time-dependent expression of basic fibroblast growth factor mRNA in skin and cerebrum is useful for detection of wound age.

##### 2.4.2.2. Diagnosis of disease and cause of death

The application of transcriptomics in specimens obtained in early postmortem period shows promising results for future studies. Miller et al. [77] aimed in their study to understand the study of prenatal human brain development and neurodevelopmental disorders, in 2014. Ramaker et al. [78] investigated brain samples of twenty-four schizophrenic, bipolar, and depressive patients, and twenty-four control cases by using RNA sequencing through which they find out the profile of postmortem transcriptome for these psychiatric illnesses. In 2019, Selley et al. [79] reviewed omics-based researches of biodiesel exhaust induced pulmonary toxicity and aimed how “healthy” is biodiesel blends instead of fossil diesel.

## 3. Advantages and novelty of the usage of omics

With the increasing use of omics methods in forensic science, it will be possible to conduct a more efficient investigation of the complicated situations that might be encountered in the future. Cooperative studies, especially in the field of proteomics, metabolomics and genomics, continue to progress in parallel to the technological developments. The excess of the obtained data and data processing are significant in this regard. In this context, cooperative studies with bioinformatics and biostatistics are essential and necessary. Nowadays, in the evaluation of forensic cases, doctors, geneticists, chemists, biologists and toxicologists work in a multidisciplinary manner; they analyze, evaluate and interpret and reconstruct the big picture together.

The use of conventional methods in forensic sciences, particularly in analytical procedures, in the new age decreases over time, by invention and presentation of each novel method. Studies should be carried out to develop new generation sequencing and mass spectrometry applications in terms of sensitivity and selectivity. In the last ten years, the use of metabolomics for the determination of drug toxicity, epigenomic studies to understand molecular mechanisms such as sudden cardiac death and identifying new biomarkers for drug toxicity are a few examples of the new era studies about omics. Finally, the more studies on this topic, the sooner it will enter the medico-legal system for justice [80, 81]. Use of omics approach and related methods, in forensic medicine, is summarized in Table.

**Table:** Use of omics approach and related methods in forensic medicine.

Omics	Methods	References
Metabolomics		
Postmortem interval	NMR-based metabolic profiling	Hirakawa et al. [21]
	MS-based metabolic profiling	Brockbals et al. [14]Dai et al. [22]Wu et al. [23]Du et al. [24]Kaszynski et al. [25]Sato et al. [26]Kang et al. [27]
Intoxication and abuse of drugs	MS-based metabolic profiling	Kim et al. [7]Shima et al. [29] Tsai et al. [5]Krumbiegel et al. [30]
Diagnosis of disease and cause of death	NMR-based metabolic profiling	Ghauri et al. [38]
	MS-based metabolic profiling	Hu et al. [37]
Proteomics		
Postmortem interval	SDS-PAGE, Western blot	Pittner at al. [44]Pittner et al. [45]
	MS-based proteomics	Choi et al. [46]Handke et al. [47]Procopio et al. [49]Prieto-Bonete et al. [50]
Intoxication and abuse of drugs	MS-based proteomics	Bittencourt et al. [51]
Diagnosis of disease and cause of death	MS-based proteomics	Broadbelt at al. [53]Palmiere et al. [54]
	Immunohistochemistry	Unkrig et al. [55]Mustafa et al. [56]Fineschi et al. [57]Edston and van Hage-Hamsten [58]
Genomics		
Detection of the biological specimens	PCR	Nakanishi et al. [12]
Postmortem interval	PCR	Pechal et al. [19]
Intoxication and abuse of drugs	PCR	Sallaee et al. [62]Ikematsu et al. [63]
Diagnosis of disease and cause of death	PCR	Tester et al. [67]Olivieri et al. [70]Martinez et al. [71]van Hylckama Vlieg et al. [72]de Haan et al. [73]
Transcriptomics		
Postmortem interval	PCR	Maeda et al. [74]Oehmichen et al. [75]Takamiya et al. [76]
Diagnosis of disease and cause of death	PCR	Miller et al. [77]Ramaker et al. [78]

## 4. Conclusion

Metabolomics, proteomics, genomics, transcriptomics, and theirs subdivisions interact with each other. This close relationship among the omics provides more reliable and useful information when they used in combination of data achieved from least two omics. These novel approaches in forensic medicine and sciences are of high importance to promote and reinforce evidence-based evaluation of medico-legal questions in trauma cases, crime scene related issues, the cause, mechanism and the manner of death. Further studies need to be performed to incorporate into routine medico-legal applications and death assessment as well as autopsy cases.

## Disclaimers/Conflict of interest

No funds were received in support of this work. This study was presented in Taiwan-Turkey Science Summit that was held in Ankara, Turkey, on 1–4 April 2018.

## Informed consent

There is no need informed consent about this work.

This study was presented at the Taiwan-Turkey Science Summit entitled “Translation of Cells, Nanomaterials and Signaling Molecules into Regenerative Medicine” between April 1 to 3, 2018.
